# Using Computer Simulations for Investigating a Sex Education Intervention: An Exploratory Study

**DOI:** 10.2196/games.6598

**Published:** 2017-05-03

**Authors:** Anastasia Eleftheriou, Seth Bullock, Cynthia A Graham, Roger Ingham

**Affiliations:** ^1^ Institute for Complex Systems Simulation Electronics and Computer Science University of Southampton Southampton United Kingdom; ^2^ Department of Computer Science University of Bristol Bristol United Kingdom; ^3^ Centre for Sexual Health Research Department of Psychology University of Southampton Southampton United Kingdom

**Keywords:** sex education, personality, STI, gender, computer simulation

## Abstract

**Background:**

Sexually transmitted infections (STIs) are ongoing concerns. The best method for preventing the transmission of these infections is the correct and consistent use of condoms. Few studies have explored the use of games in interventions for increasing condom use by challenging the false sense of security associated with judging the presence of an STI based on attractiveness.

**Objectives:**

The primary purpose of this study was to explore the potential use of computer simulation as a serious game for sex education. Specific aims were to (1) study the influence of a newly designed serious game on self-rated confidence for assessing STI risk and (2) examine whether this varied by gender, age, and scores on sexuality-related personality trait measures.

**Methods:**

This paper undertook a Web-based questionnaire study employing between and within subject analyses. A Web-based platform hosted in the United Kingdom was used to deliver male and female stimuli (facial photographs) and collect data. A convenience sample group of 66 participants (64%, 42/66) male, mean age 22.5 years) completed the *Term on the Tides,* a computer simulation developed for this study. Participants also completed questionnaires on demographics, sexual preferences, sexual risk evaluations, the Sexual Sensation Seeking Scale (SSS), and the Sexual Inhibition Subscale 2 (SIS2) of the *Sexual Inhibition/Sexual Excitation Scales-Short Form (SIS/SES - SF)*.

**Results:**

The overall confidence of participants to evaluate sexual risks reduced after playing the game (*P*<.005). Age and personality trait measures did not predict the change in confidence of evaluating risk. Women demonstrated larger shifts in confidence than did men (*P*=.03).

**Conclusions:**

This study extends the literature by investigating the potential of computer simulations as a serious game for sex education. Engaging in the *Term on the Tides* game had an impact on participants’ confidence in evaluating sexual risks.

## Introduction

### Background

Sexually transmitted infections (STI), including human immunodeficiency virus (HIV) are ongoing concerns. Rates of new STI diagnoses are increasing in most countries of the world, particularly among young people [1]. The best method for preventing the spread of these infections is the correct and consistent use of condoms [2,3]. However, people continue to engage in risky sexual behaviors, such as having condomless sex [4] and using condoms incorrectly [5,6]. Given these threats to individual and public health, many strategies and intervention programs have been developed to encourage consistent and correct condom use; these interventions have met with varied levels of success [7].

There are several reasons for variation in the impact of interventions. First, a “one-size-fits-all” approach is unlikely to be effective with all the intended recipients, given that they will vary in age, sexual preferences, sexual experience, and sexual attitudes [8-10]. Kirby and Laris [9] noted that it is important for a sex education program to meet the needs of the audience, taking into account different backgrounds and community values. Recent research has shown that personality traits, as well as perceived attractiveness, can significantly affect the perception of sexual risk [11]. Henderson et al [12] demonstrated how individuals high in Sensation Seeking (SS), compared with those lower in SS, rate potential partners as more attractive and are more willing to have sex with those partners, but they feel that they are less likely to contract an STI. Results of a similar study showed that participants who were engaged in a wider range of potentially risky sexual behaviors were characterized by higher Sexual Sensation Seeking Scale (SSS) [13]. These results support the idea that sex education programs may benefit from the inclusion of components aimed at teaching young people to satisfy their preferences for SSS through sexual behaviors involving minimum risk.

In addition to SSS, another measure that might be relevant is Sexual Inhibition (SI), particularly a subscale from the *Sexual Inhibition/Sexual Excitation Scales (SIS/SES*), which assesses sexual inhibition due to performance consequences (Sexual Inhibition Subscale 2, SIS2). In a sample of gay men, Bancroft et al [14] showed that condomless anal sex was more likely among those who scored lower on sexual inhibition due to the “threat of performance consequences” (eg, threat of an STI). In samples of both heterosexual men and women, research has supported the association between scores on SIS2 and sexual risk taking behavior [15,16].

Second, some people feel less vulnerable to STIs based on their belief that they can ascertain whether a potential partner is likely to be infected or not on the basis of the way that they look or some other superficial characteristic. It has been shown that men feel that they would be able to make judgments about other people’s sexual health status based on perceived attractiveness [8,17]. In one study, participants believed that judgments of sexual unfaithfulness could be made of the face alone, without consideration of behavioral cues [18]. Hence, one main aim of sex education programs, but one that has been seldom addressed in interventions to date, should be to challenge this false sense of security.

Third, many sex education programs have been described, particularly by young people, as being “boring” or “irrelevant” to their needs [19]. Carswell et al [20] emphasized the importance of Web-based sex education interventions, pointing out how attractive they are for young people, as they offer a confidential and convenient medium for accessing health information, avoiding the embarrassment of discussions with teachers and health providers, and overcoming potential boredom by using an interesting game format. DeSmet et al [21] and D’Cruz et al [22] highlighted the importance of certain game design features that should be considered when developing a game for sex education, such as individual tailoring, goal-setting, narrative or story, audiovisual effects, interactivity, challenge on different levels, rewards, and immediate feedback.

Bearing in mind these three issues, one direction in which sexual health interventions could profitably develop involves the use of serious games. As young people are very familiar with computer and video game playing [23,24], they may find it easier and more motivating to engage with this format of sex education intervention [25,26]. Serious games could focus on increasing understanding of the risks and addressing misconceptions [27] in the complex area of STI transmission. This could happen if people are given the potential to engage with a simulated world of people and their sexual interactions, in order to see how easy it is for diseases to spread. In serious games, it is predominantly the players who direct events and are therefore actively involved in the learning process [28,29], in contrast to traditional sex education interventions, where learners are generally relatively passive [30,31].

There has been some previous research exploring the use of games for sex education. *The Source* [19] is an alternative reality game that was delivered over 5 weeks to young people aged between 13 and 18 years. Each week focused on a different topic (including sexual health) that was taught using various methods, such as puzzles, board games, digital media tasks, and scavenger hunts. No behavioral outcome data were reported. However, participants enjoyed the tasks and some of them reported that *The Source* reinforced their decisions to engage in safer sex, although many of them commented that they found the board games boring and not as interactive as the computerized tasks.

Verran et al [32] explored the idea of using a computer simulation called *SimZombie* for educational activities about the epidemiology of an infectious disease (albeit not a sexually transmitted one) carried out at the Manchester Science Festival 2011. *SimZombie* makes use of the fact that many young people show interest in zombies and therefore it helps them engage more than they would do with a “one-way” mode of communication, such as a leaflet explaining the epidemiology of diseases. In the activities designed by Verran and colleagues, 10 teams of 4-6 participants (predominantly families or teenagers) had to answer 3 rounds of questions about monsters, microbiology, and general scientific knowledge. After each round, their answers were marked by being inserted into the simulation. Enthusiastic feedback given by participants evidenced learning through these activities.

Shegog et al [33] developed a stand-alone Web-based game of 13 lessons, called *It’s Your Game (IYG)*. IYG lessons, which target early adolescents, include activities like interactive 2D exercises, quizzes, animations, and peer video. An evaluation of this game [34] showed no significant difference in the delay of sexual activity between intervention and control students; however, there was a significant positive between-group difference on psychological variables related to STI and condom knowledge, perceived norms about sex and condom use self-efficacy.

Although previous studies have suggested potential positive benefits of serious games in health education, very little research has been carried out to investigate the influence of computer simulations for sex education specifically. As it is possible that the benefits of such games will vary according to age [35], gender [19], and sexual attitudes [8], it would be useful to investigate the possible impact of these variables.

### Aim of This Study

The primary purpose of this study was to explore the potential of computer simulation as a serious game for sex education and how the effects of a serious game might be moderated by personality traits, age, and gender. The research questions were (1) Do gender, age, and personality traits influence levels of confidence in evaluating sexual risk? (2) Does a simulation in the form of a serious game influence participants’ confidence regarding the assessment of sexual risk? and (3) Do gender, age, and personality traits influence the impact of the serious game in altering participants’ confidence in evaluating sexual risks?

## Methods

### Sample and Recruitment

Men and women in Southampton and surrounding areas were recruited via social media (Facebook, Twitter), posters at the University, and community advertisement boards. Potential participants were informed that data would be collected using an electronic quiz in order to investigate the use of a serious game in the form of a computer simulation for sex education. The posters contained the following information: “I would like to see how you will perform in a game we have developed for sex education.” Inclusion criteria were 18-30 years of age and English speaking. A total of 42 men, 22 women, and 2 participants who chose “other” for the question on gender were screened and all met the inclusion criteria.

Data were collected in May 2016. In total, 22 participants completed the experiment online at home, with a further 44 doing so in the lab. All participants were provided with a study information sheet and indicated electronic informed consent. The study took approximately 25 min.

### Study Design

This was a Web-based questionnaire study (that used between and within subject analyses). The study employed a quiz to collect data. A draft quiz was initially trialed on 6 pilot study participants and was then refined on the basis of their feedback during individual “think aloud” sessions. “Think aloud” is a commonly used protocol for usability testing of an intervention [36].

### Measures

The final questionnaire comprised four sections: (1) demographic information, (2) the participant’s sexual risk evaluations, (3 personality trait questionnaires (SSS [37] and SIS2 of the *SIS/SES – Short Form* [38]), and (4) the *Term on the Tides* quiz. The order of the 10 test questions in the quiz was fully randomized for each participant.

#### Demographics and Sexual Behavior

Participants were asked about their age, ethnicity, gender, and sexual orientation. Ethnicity options included white, black, Asian, mixed, and other. Gender options were “male,” “female,” “other,” and “prefer not to say,” and for sexual orientation (preference), “men,” “women,” “both,” or “none.”

#### Personality Traits Questionnaires

The SSS [37] assesses the tendency to seek out varied, novel, and complex sexual experiences and the desire to take personal, physical, and social risks in order to enhance sexual sensations. A sample item is “I am interested in trying out new sexual experiences.” The SSS can be used with both men and women, and shows good construct validity and internal consistency (Cronbach alpha=.83 for men and Cronbach alpha=.81 for women) [37]. Questions were answered on a 4-point scale, ranging from 1 (*not at all like me*) to 4 (*very much like me*). The sums of the scores are calculated to produce a total score on SSS, with a higher score indicating higher levels of the trait.

The SIS2 assesses individual propensity to inhibit arousal because of threat of performance consequences (such as contracting an STI) [38]. This scale is one of three subscales of the *SIS/SES – SF*. A sample item is “If I realize there is a risk of catching a sexually transmitted disease, I am unlikely to stay sexually aroused.” SIS/SES-SF can be used with both men and women, shows good construct validity and test-retest reliability [38]. Response options range from 1 (*strongly disagree*) to 4 (*strongly agree*); after suitable recoding, scores are summed to produce a total score, with a higher score indicating higher levels of inhibition.

#### Evaluation of Sexual Risk

Participants were asked to respond to the following statement: “Risks taken during unprotected sex are easy to evaluate.” Response options ranged from 1 (*strongly agree*) to 5 (*strongly disagree*). This item was used as a measure of the participants’ confidence in evaluating sexual risk.

Participants also rated their level of agreement with this statement: “The risk that someone takes when they have unprotected sex depends on the risk taking behavior of the other people in the sexual population.” Response options ranged from 1 (*strongly agree*) to 5 (*strongly disagree*). This item was included to assess the extent to which participants felt that they were in control of potential risky situations.

Both items were completed before (*t*_1_), and immediately after (*t*_2_), completion of the *Term on the Tides* quiz.

#### Game Description: Term on the Tides

The quiz concerns a cruise called *Term on the Tides*, developed for this study, where the user of the game is asked to answer some questions about the sexual health status of people on the cruise, at different stages of the simulation (developed in Java).

The storyboard was introduced with the following: “You embarked on a singles love cruise sailing from Mykonos down to Ibiza. The ship is full of heterosexual single men and women who are looking for easy, no-strings attached sexual encounters with each other. Passengers have not been medically examined and therefore are unaware of whether are carrying a sexually transmitted disease or not. The journey time to your destination is 1 week. The ship is fully prepared for any lengthy journey and it is well-stocked with food and supplies including an inexhaustive supply of condoms. Due to the nature of the cruise, everyone is unconcerned with forming a relationship. So whether they will choose to have sex with someone, with or without a condom, is purely based on physical appearance.” The main task of the participants was to give the right answer to 10 questions or scenarios regarding the sexual health status of certain people on the cruise.

**Figure 1:**
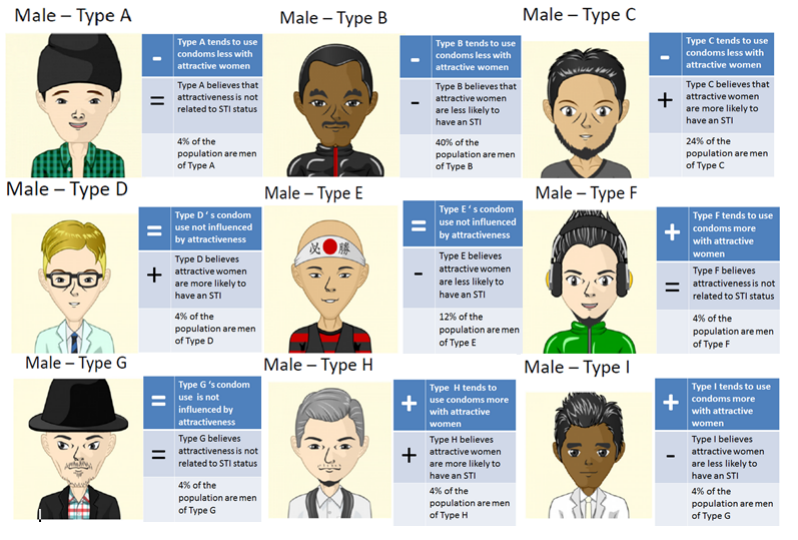
Male profiles in the computer simulation. Each type has two symbols to describe his personality. For the top one, a “+” indicates that a person uses condoms more with women that they find attractive, a “−” indicates that a person uses condoms less with women that they find attractive, and an “=” indicates that condom use is not affected by attractiveness. The bottom symbol represents the belief of a person with regards to the relationship between sexually transmitted infection (STI) risk and attractiveness: “+” means the person believes that attractive women are more likely to have an STI, “−” means that they believe attractive women are less likely to have an STI, and “=” means that the person believes that attractiveness is not related to STI.

The scenarios presented in the questions were based on the responses of male participants in a previous study [8], regarding their reported condom use intentions according to their perceptions of women’s attractiveness. These responses were used in order to produce the profiles of the people in the simulation (Figure 1). Each person’s profile had two characteristics: (1) how their condom use intentions and their judgments of STI likelihood varied with the attractiveness of a potential sexual partner, and (2) how the STI likelihood judgments of the person varied with the attractiveness of a potential sexual partner. For example, the Type A man shown in Figure 1 tends to use condoms less with women he finds more attractive (therefore he gets a “−” sign in the first box of his profile) and also believes that STI status is not associated with perceived attractiveness (therefore he gets a “=” in the second box of his profile). As nine different profiles could be created using combinations of the three symbols (“+,” “−,” “=”), nine different types of men were created and several copies (clones, ie, people with similar behavior) of those were included in the simulation. The number of clones of each type used was proportional to the number of participants in the first study [8] who fitted those types, based on their responses. In total, there were 100 men in the simulation.

**Figure 2:**
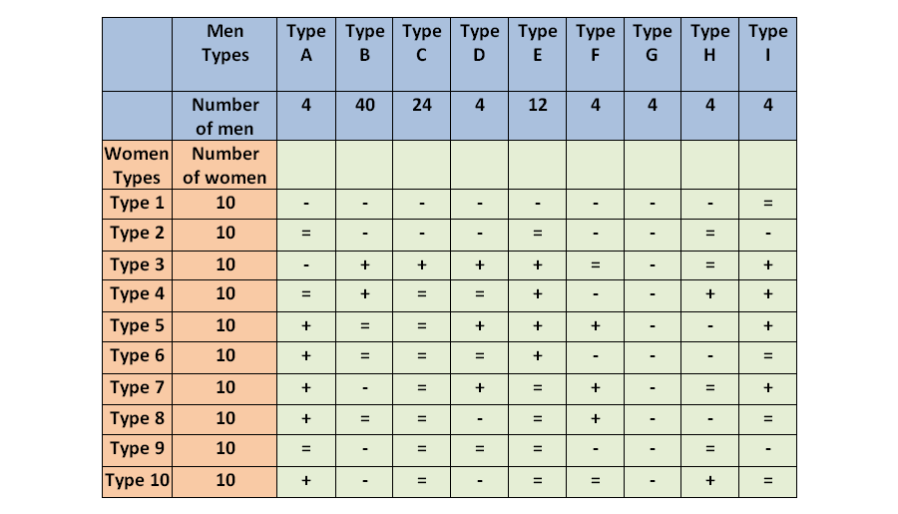
Attractiveness table. A “+” indicates that the specified man is attracted to the specified woman, a “−” indicates that the man specified is not attracted to the specified woman, and an “=” means that the man does not find the woman either attractive or unattractive.

A summary of attractiveness ratings given by each man in the previous study to each woman was shown to the users throughout the game (Figure 2). The profiles of the women were chosen in a similar way to that described above for men, with the difference being that we constructed the female profiles based on how men rated female pictures in the first study [8]. Ten types of women were chosen and we tried to include as much variability in attractiveness and STI ratings as possible. Ten clones of each one of those profiles was included in the simulation, leading to a total of 100 simulated women.

**Figure 3:**
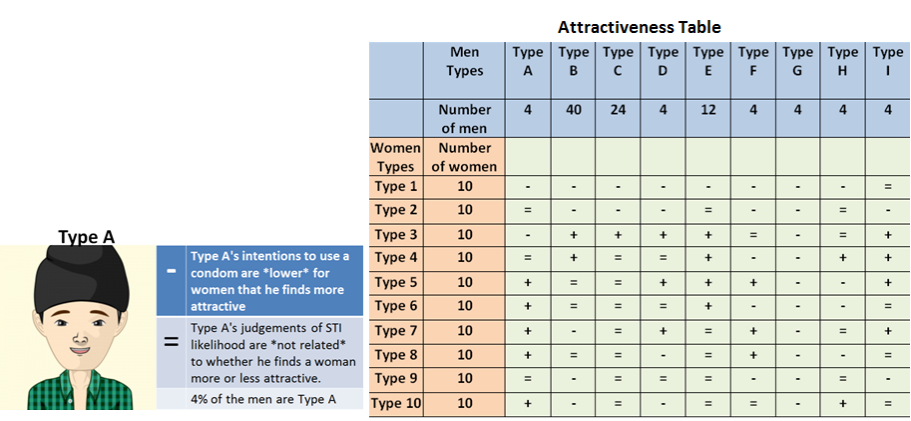
Example question: Is a type A man more likely to get an infection from a Type 4 woman or a Type 7 woman?

There were various different framings used and each one of them clearly specified the precise situation of the people in the simulation. For example, in one, the user is asked to predict the outcome of an encounter between a man of Type A and a Type 4 woman versus an encounter between a man of Type A and a Type 7 woman (see Figure 3). When participants were able to correctly judge which sexual interaction was most risky, they were awarded 1 point. Ten scenarios were presented; therefore, the score for someone who did not make any correct estimates would be 0 and for someone who accurately answered all scenarios would be 10.

**Figure 4:**
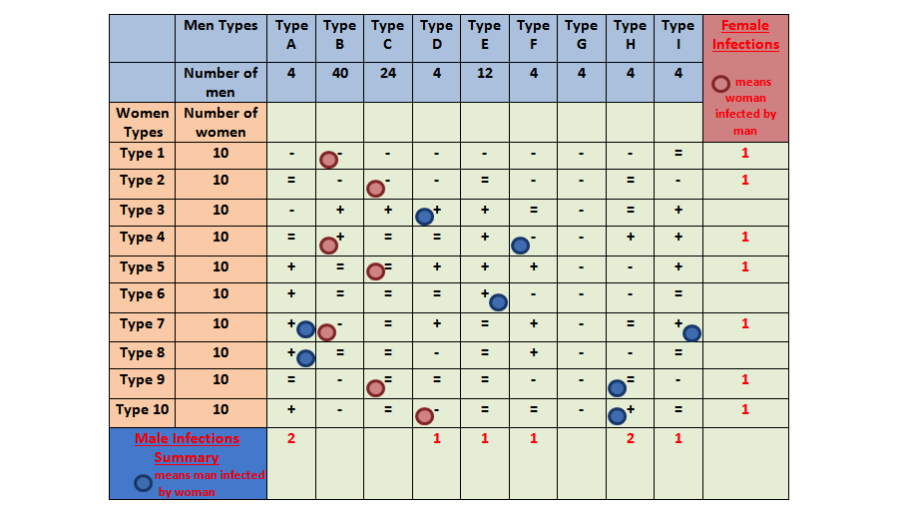
Feedback given to the participants.

The final stage of the game was the feedback provided to the users. Users watched a series of encounters between men and women in the simulated population, and they received information on how well they managed to estimate risk in each scenario, by receiving an overall score for the quiz and appropriate feedback to each question (see Figure 4).

In order to determine the correct answers to the questions, the computer simulation makes use of the attractiveness and condom use intentions of each person on the cruise. At the beginning of each simulation, infections are allocated to the population at random. People have the chance to meet each other and decide (1) whether to have sex or not, and, if they decide to have sex, (2) whether to have sex with or without a condom, based on the variables of attractiveness and condom use intentions specified for their type. There is a very high chance of an STI transmission when someone has condomless sex with another person who carries an infection. An average over 100 simulations was used for this quiz.

In order to account for possible biases stemming from the appearance of the images used for each type of person in the game, a random selection of pictures was allocated at the beginning of the game, from a selection of three different versions (white, black, and Asian faces).

### Procedure

After providing informed consent, each participant completed the self-administered questionnaires followed by the quiz. A £100 Amazon voucher was offered as an incentive to the person with the highest score on the quiz. The Ethics Committee of the University of Southampton approved the study.

### Data Analysis

To identify factors influencing the confidence ratings and the levels of change of confidence of evaluating sexual risk, a series of bivariate associations (Pearson correlation coefficients) and independent *t* test were conducted between the main variables examined (age, gender, personality traits, quiz score, and confidence of evaluating sexual risk before and after the game). Matched pairs *t* test was used to test whether participants’ confidence in evaluating STI risk changed from *t*_1_ to *t*_2_, that is, before and after the simulation.

## Results

### Descriptive Statistics

#### Demographics

A total of 66 participants (42 men, 22 women, and 2 “other”) had a mean age of 22.5 years (SD 3.3, min 18, max 29). The majority of participants were identified as white (80%, 53/66) and as heterosexual (approximately 88% [58/66]; see Table 1).

#### Personality Variables

On the SSS, the mean score for men was 23.1 and for women was 22.5. Higher scores indicated greater sexual sensation seeking. There was no significant gender difference in this measure (*t*_62_=0.42, not significant [ns]).

**Table 1:** Sample demographics.

Variable		n
**Ethnicity**		
	White	53
	Black	3
	Asian	7
	Mixed	1
	Other	2
**Gender**		
	Men	42
	Women	22
	Prefer not to say	2
**Age (in years)**		
	18-24	46
	25-30	20

On the SIS2, the higher a participant scored, the higher the propensity for sexual arousal to be reduced in the face of threats of performance consequences. The mean SIS2 score for women (12.9) was significantly higher than that for men (11.2) (*t*_62_=3.05, *P*<.005).

### Research Question 1: Do Gender, Age, and Personality Traits Influence Levels of Confidence in Evaluating Sexual Risk?

At *t*_1_, women believed that risk was easier to assess than did men, with the mean ratings 3.82 and 3.25, respectively (*t*_62_=1.99, *P*=.05); however, the belief that risk depends on the other people did not differ between men and women (*t*_62_=0.19, ns).

Age showed no significant correlations with participants’ confidence in evaluating risk (*r*=.11, n=66, ns) or their belief that risk depends on others in the population (*r*=−.05, n=66, ns).

At *t*_1_, no significant correlations between SSS or SIS2 and participants’ confidence in evaluating sexual risk (*r*=.15 and *r*=.10, respectively, n=66, ns) or in believing that the sexual risk depends on others in the population (*r*=.15 and *r*=.13, respectively, n=66, ns) were found.

### Research Question 2: Does a Simulation in the Form of a Serious Game Influence Participants’ Confidence Regarding the Assessment of Sexual Risk?

In response to the statement “Risks taken during unprotected sex are easy to evaluate,” the mean score before the game was 3.47, and after the game it was 2.98. A matched pairs *t* test showed that the change in confidence was significant (*t*_63_=5.81, *P*<.001). Before the intervention, 56% agreed that it was easy to evaluate risk, whereas only 44% did so after the intervention. Similarly, 24% disagreed before the game compared with 38% after the game.

In response to the statement “The risk that someone takes when they have unprotected sex depends on the risk taking behavior of the other people in the sexual population,” the mean score before the game was 3.74, and after the game it was 3.77. A matched pairs *t* test revealed that the difference between these mean scores was not significant (*t*_65_=0.27, ns).

### Research Question 3: Do Gender, Age, and Personality Traits Influence the Impact of the Serious Game in Altering Participants’ Confidence in Evaluating Sexual Risks?

There was a significant gender difference in the impact of the game on confidence ratings; women had a greater reduction in confidence regarding their perceived ability to evaluate sexual risk than did men (mean change scores for men 0.30 and 0.82 for women; *t*_60_=3.11, *P*<.005). There were no gender differences in change scores for believing that risk depends on other people (mean change scores for men 0.05 and −0.18 for women; *t*_62_=0.92, ns). Age did not correlate with either of the risk measures (for easy, *r*=.12, n=64, ns, and for risk depends on others, *r*=−.18, n=60, ns).

Similarly, there was no correlation between the changes in confidence ratings concerning assessment of sexual risk before and after the game, and scores on SSS or SIS2 (*r*=−.06, n=64, ns and *r*=.11, n=64, ns, respectively). Finally, there was also no correlation between the changes in confidence ratings concerning sexual risk depending on others before and after the game, and scores on SSS or SIS2 (*r*=.20, n=66, ns, and *r*=−.03, n=66, ns, respectively).

### Additional Results on Quiz Scores

The average score on the quiz across the 66 participants was 5 out of 10 (min=2, max=8; the mean for men was 5.1, and 4.8 for women; *t*_62_=0.79, ns). There were no differences in scores according to age (*r*=−.13, n=66, ns) or whether participants completed the study at home or in the laboratory (*r*=.20, n=66, ns). Anecdotal reports after the study indicated that many participants found the game very interesting and thought provoking, but also quite challenging.

No significant correlation was found between scores on the quiz and confidence in evaluating sexual risk at *t*_1_ (*r*=−.06, n=66, ns and *r*=−.07, n=66, ns, for risk for self and risk for others, respectively), or the change in confidence regarding risk-assessment between *t*_1_ and *t*_2_ (*r*=−.01, n=64, ns and *r*=−.06, n=66, ns, respectively).

## Discussion

### Principal Findings

This study sheds some light on the use of computer simulations as a serious game for sex education. There was a significant change in participants’ confidence in evaluating sexual risk in the *Term on the Tides* game. Before they played the game, the majority of the participants believed that it was easy to evaluate the risks of unprotected sex. The serious game challenged individuals’ confidence to evaluate risks and, as a result of this, approximately 40% of participants reported lower confidence after playing the game than they did at the *t*_1_ baseline. The fact that overall confidence in evaluating risks reduced after the participants had engaged with the game illustrates a potentially positive public health outcome. It would be expected that lower confidence in evaluating sexual risks would lead to greater caution in sexual encounters.

Age and the personality trait variables—SSS and SIS2—were not correlated with the confidence of evaluating risk or with the level of change in confidence before and after the game. Gender, however, did have an effect, as women demonstrated a bigger shift in confidence of evaluating sexual risk than men. This finding agrees with a previous study on *The Source*, an alternative reality game [19], which suggested that women were influenced more by engaging in the game than men. Brüll et al [39] argued that males prefer the use of more explicit terminology to describe sexual activity in a game than females.

Previous studies have shown that the difficulty of a game is a major determinant of the influence that it has on users, mainly because users get discouraged if the game is very difficult or they get bored if it is too easy [40,41]. Although in this case participants were not asked directly to comment on the difficulty of the game, we observed that many reflected on the experience and discussed with the researcher what they had learned from the game. Most of them found it “challenging,” and may have been motivated to continue because the person with the highest score would win an Amazon voucher.

Future research should investigate the effect of age on the influence of a sex education game using a bigger sample, as there were not enough older participants in this study to report findings regarding this variable with confidence. Additionally, the relationship status and relationship power of the participants should be investigated, as this might significantly change the way they associate with the characters of the game and therefore their evaluation of sexual risk [42]. Moreover, different ways to enhance immersion in the game should be examined, in order to keep the interest of the users high and keep them engaged with the educational activity for as long as possible; for example, by using a virtual reality (360) simulation, which will challenge the users’ sexual health knowledge and attitudes on various difficulty levels using a somewhat less artificial and sterile environment or characters [43]. Sexual arousal during the sex education game could also be investigated as it is a factor that influences condom use in real-life contexts [44].

This study is a step toward the design of tailored and relevant sex education interventions, as called for by DeSmet [21] and D’Cruz [22]. Although this study includes several features recommended by these authors, for example, goal-setting, narrative, and so on, it might be profitable to explore greater interactivity and the use of audiovisual stimuli.

### Strengths and Limitations

Some limitations of the study need to be acknowledged. Participants were not asked systematically about the difficulty of the game and therefore we only have anecdotal information about this variable. Also, we used a relatively small convenience sample and no behavioral outcomes or behavioral theory were assessed. Notwithstanding these limitations, this study is the first to explore the influence of computer simulations in the form of a serious game for sex education in relation to risk perception, and to investigate the impact that individual difference variables (age, gender, and personality) may have on the outcome. The results would be particularly useful for serious games designers for sex education as they provide some limited but promising insight into which aspects of games-tailoring could be beneficial and worth investigating further.

### Conclusions

Computer simulations, presented in the form of a serious game, had an impact on participants’ confidence in evaluating sexual risk, especially for women. This suggests that serious games developed for use in this setting should be further investigated and perhaps gender-tailored. Working toward these goals might contribute to a reduction in STI rates. Personality traits and age were not related to the change in participants’ confidence in evaluating sexual risks before and after engaging in the game.

## Abbreviations

HIV: human immunodeficiency virus
IYG: It’s Your Game
SES: Sexual Excitation Scale
SI: Sexual Inhibition
SIS2: Sexual Inhibition Subscale 2
SS: Sensation Seeking
SSS: Sexual Sensation Seeking Scale
STI: sexually transmitted infection
